# Incorporating Distant Sequence Features and Radial Basis Function Networks to Identify Ubiquitin Conjugation Sites

**DOI:** 10.1371/journal.pone.0017331

**Published:** 2011-03-09

**Authors:** Tzong-Yi Lee, Shu-An Chen, Hsin-Yi Hung, Yu-Yen Ou

**Affiliations:** Department of Computer Science and Engineering, Yuan Ze University, Chung-Li, Taiwan; University of South Florida College of Medicine, United States of America

## Abstract

Ubiquitin (Ub) is a small protein that consists of 76 amino acids about 8.5 kDa. In ubiquitin conjugation, the ubiquitin is majorly conjugated on the lysine residue of protein by Ub-ligating (E3) enzymes. Three major enzymes participate in ubiquitin conjugation. They are – E1, E2 and E3 which are responsible for activating, conjugating and ligating ubiquitin, respectively. Ubiquitin conjugation in eukaryotes is an important mechanism of the proteasome-mediated degradation of a protein and regulating the activity of transcription factors. Motivated by the importance of ubiquitin conjugation in biological processes, this investigation develops a method, UbSite, which uses utilizes an efficient radial basis function (RBF) network to identify protein ubiquitin conjugation (ubiquitylation) sites. This work not only investigates the amino acid composition but also the structural characteristics, physicochemical properties, and evolutionary information of amino acids around ubiquitylation (Ub) sites. With reference to the pathway of ubiquitin conjugation, the substrate sites for E3 recognition, which are distant from ubiquitylation sites, are investigated. The measurement of F-score in a large window size (−20∼+20) revealed a statistically significant amino acid composition and position-specific scoring matrix (evolutionary information), which are mainly located distant from Ub sites. The distant information can be used effectively to differentiate Ub sites from non-Ub sites. As determined by five-fold cross-validation, the model that was trained using the combination of amino acid composition and evolutionary information performs best in identifying ubiquitin conjugation sites. The prediction sensitivity, specificity, and accuracy are 65.5%, 74.8%, and 74.5%, respectively. Although the amino acid sequences around the ubiquitin conjugation sites do not contain conserved motifs, the cross-validation result indicates that the integration of distant sequence features of Ub sites can improve predictive performance. Additionally, the independent test demonstrates that the proposed method can outperform other ubiquitylation prediction tools.

## Introduction

Ubiquitin (Ub) is a small protein that consists of 76 amino acids about 8.5 kDa. Ubiquitin conjugation sites of protein (Ubiquitylation), which is an essential post-translational modification, is a sequential process that involves a group of enzymes known as E1 (activating enzyme), E2 (conjugating enzyme) and E3 (ubiquitin ligase). The ubiquitylation system is well-known for the selective degradation of serveral short-lived proteins in eukaryotic cells [1]. The attachment of a ubiquitin or poly-ubiquitin chains to proteins influences serveral cellular processes, including transcriptional regulation, signal transduction, development, apoptosis, endocytosis, and cell proliferation [2]. Ubiquitin is mostly conjugated on the lysine residue of a protein by Ub-ligating (E3) enzymes. The E3 ligase must be sufficiently specific and must act only on a defined subset of cellular targets to ensure signal fidelity [3]. Another enzyme, E4, has that can stabilize and extend a poly-ubiquitin chain, has also been found [4].

With the development of high-throughput tandem mass spectrometry-based proteomics, the number of studies of the comprehensive identification ubiquitylated proteins and their conjugated sites is increasing [5]. UbiProt [6] identified all experimentally verified ubiquitin-conjugated sites from the publicly literature. Some of the entries include information about enzyme data obtained by enzyme purification and isolation. The entries supply annotations of the ubiquitin-conjugated and the exact positions of the ubiquitin-conjugation sites. UniProtKB/Swiss-Prot [7] is a comprehensively annotated protein database. Both experimentally validated and putative ubiquitin-conjugated annotations can be obtained from the post-translation modification annotations in the database.

Experimental identifications of ubiquitin-conjugation sites on ubiquitylated proteins *in vivo* and *in vitro* are the foundation for understanding the mechanisms of ubiquitination dynamics. However, these experiments are commonly time-consuming, labor-intensive and expensive. the *in silico* prediction of ubiquitin-conjugated sites with high predictive performance could be promising for preliminary analyses and could greatly reduce the number of potential targets that require further *in vivo* or *in vitro* confirmation. UbiPred [8] used an algorithm for mining informative physicochemical properties from protein sequences to train SVM-based ubiquitylation site prediction system. Based on leave-one-out cross-validation, the SVM model that is trained with 31 physicochemical properties was evaluated. It was found to improve the predictive accuracy from 72.1% to 84.4%. Recently, Radivojac *et al.* [9] have investigated that the sequence biases and structural preferences around known ubiquitination sites are similar to those of intrinsically disordered protein regions. Additionally, Radivojac *et al.* developed a random forest predictor of ubiquitination sites, UbPred, that could reach a balanced accuracy of 72%.

Given the importance of ubiquitin conjugation in biological processes, this investigation presents a method, UbSite, in which an efficient radial basis function (RBF) network is utilized to identify protein ubiquitin conjugation (ubiquitylation) sites. The experimentally verified ubiquitylated proteins and ubiquitylation sites are collected from UbiProt [6] and UniProtKB/Swiss-Prot [7]. Not only amino acid composition, but also structural characteristics, physicochemical properties, and evolutionary information of amino acids around the ubiquitylation (Ub) sites are investigated. With reference to the pathway of ubiquitin conjugation, which is by sequential process that involves a group of enzymes, E1 (activating enzyme), E2 (conjugating enzyme) and E3 (ubiquitin ligase), the distant sequence features of ubiquitylation sites for E3 recognition are investigated. A position specific scoring matrix (PSSM), which is generated by PSI-BLAST [10] search against a non-redundant database of protein sequences, is utilized to study the evolutionary information surround the ubiquitin conjugation sites. The constructed PSSM is regarded as a measure of residue conservation in a window of a given length. Based on the measurement of F-score in a large window size (−20∼+20), the statistically significant amino acid composition and evolutionary information, which are mainly located at positions distant from the ubiquitylation sites, can be utilized effectively to differentiate ubiquitylation sites from non-ubiquitylation sites. An evaluation of the trained models based on five-fold cross-validation revealed, that the prediction sensitivity, specificity and accuracy were 65.5%, 74.8%, and 74.5%, respectively. The independent test demonstrates shows that UbSite outperform previous ubiquitylation prediction tools.

## Materials and Methods

**Figure 1:**
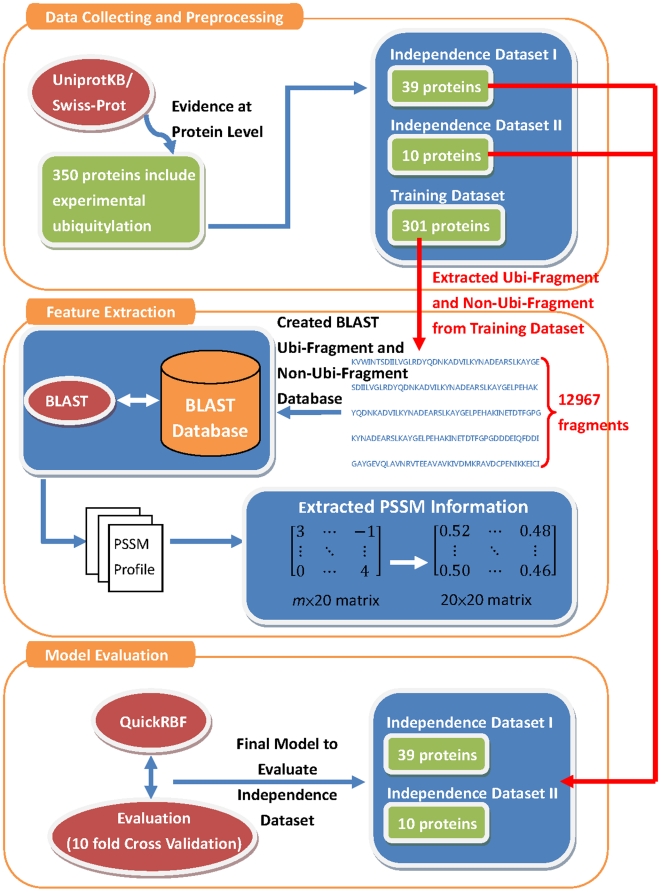
The analytic flowchart of UbSite.

As shown in Fig. 1, the proposed approach, UbSite, is composed of three major analytical steps - data collection and preprocessing, feature extraction, and model training and evaluation. This investigation comprehensively analyzes the structural characteristics and physicochemical properties that surround the ubiquitin conjugation sites. The details of the analysis are described as follows.

### Data collection and preprocessing

Experimentally confirmed ubiquitin conjugation sites are collected from UbiProt [6] and UniProtKB/Swiss-Prot [7]. UbiProt consists of 158 experimentally confirmed ubiquitin-conjugation sites. Then, we extracted the sequences from release 57.0 of UniProtKB/Swiss-Prot if the sequences are annotated as ‘ubiquitin’ in the ‘MOD_RES’ fields. We also removed the sites that are annotated as “by similarity”, “potential” or “probable”. A total of 337 entries are annotated as ubiquitin-conjugated proteins in UniProtKB/Swiss-Prot, and they include 416 ubiquitylation sites. After removing the redundant data from UbiProt and UniProtKB/Swiss-Prot, a total of 442 experimental ubiquitylation sites associated with 350 ubiquitylated proteins are obtained. In this work, the 442 experimental ubiquitylation sites are regarded as the positive dataset.

To prevent overestimation of the predictive performance, homologous sequences are removed from the training data by using a window size of 2*n*+1 for ubiquitylation sites. With reference to the reduction process in MASA [11], two ubiquitylated protein sequences with more than 30% identity were defined as homologous sequences. Then, two homologous sequences were specified to re-align the fragment sequences using a window length of 2*n*+1, centered on the ubiquitylation sites using BL2SEQ [12]. For two fragment sequences with 100% identity, when the ubiquitylation sites in the two proteins are in the same positions, only one site was kept. The homologous negative data were also reduced by using the same approach.

With respect to classification, the predictive performance of the trained models may be overestimated because of the over-fitting of a training set. To estimate the real predictive performance, the experimental ubiquitylation sites, whose annotated dates are after April 4 2006, are selected as the independent test set. As shown in Table 1, the data in the non-homologous training set include 385 positive sites (ubiquitylation) and 12582 negative sites (non-ubiquitylation) in 301 ubiquitylated proteins. The data of the non-homologous independent test set include 57 positive sites and 3502 negative sites in 49 ubiquitylated proteins. Following the evaluation by five-fold cross-validation, the trained model with the highest accuracy was further evaluated based on independent test data. The independent test sets were utilized to test not only the proposed method but also the previously proposed ubiquitylation prediction tools, UbiPred [8] and UbPred [9].

**Table 1:** The statistics of non-homologous training data and independent test data for ubiquitylation and non-ubiquitylation sites.

	Training data^a^	Independent data^b^
Number of proteins	301	49
Number of ubiquitylated lysines	385	57
Number of non-ubiquitylated lysines	12,582	3,502

a Training data: the annotation date of experimental ubiquitylation site is before April 4 2006.

b Independent data I: the annotation date of experimental ubiquitylation site is between April 4 2006 and January 1 2008.

### Feature extraction and coding

#### Coding of amino acid sequences

Fragments of amino acids are extracted from positive and negative training sets using a window of length 2*n*+1 that is centered on ubiquitylation sites. Various values of *n* are used to determine the optimal window length. The BLOSUM62 matrix is adopted to represent the protein primary sequence information as the basic feature set for learning radial basis function networks. A matrix of *m*×*n* elements is used to represent each residue in a training dataset, where *n* denotes the window size and *m* = 21, which elements comprise 20 amino acids and one terminal signal. Each row of the normalized BLOSUM62 matrix is adopted to encode one of 20 amino acids.

#### Compositions of amino acids and amino acid pairs

A total of *n* vectors {*x_i_*, *i* = 1, …, *n*} were used, to represent all *n* proteins in the training data. Each vector is labeled with the group of its corresponding protein (e.g. ubiquitylated or non-ubiquitylated). The vector *x_i_* has 20 elements for the amino acid composition and 400 elements for the amino acid pair composition. The 20 elements specify the numbers of occurrences of 20 amino acids normalized with the total number of residues in the protein, and the 400 elements specify the numbers of occurrences of 400 amino acid pairs normalized with the total number of residues in the protein. In this investigation, amino acid composition and amino acid pair composition are combined, yielding, 420 elements in each vector.

#### Position Specific Scoring Matrix Profiles

In the point of view of structure, several amino acid residues of a protein can be mutated without changing its structure, and two proteins may have similar structures with different amino acid compositions. Position Specific Scoring Matrix (PSSM) profiles, which have been extensively utilized in protein secondary structure prediction, subcellular localization and other bioinformatics problems are adopted herein with significant improvement [13], [14], [15]. The PSSM profiles were obtained by PSI-BLAST against non-redundant fragment sequences of Ub sites.

**Figure 2:**
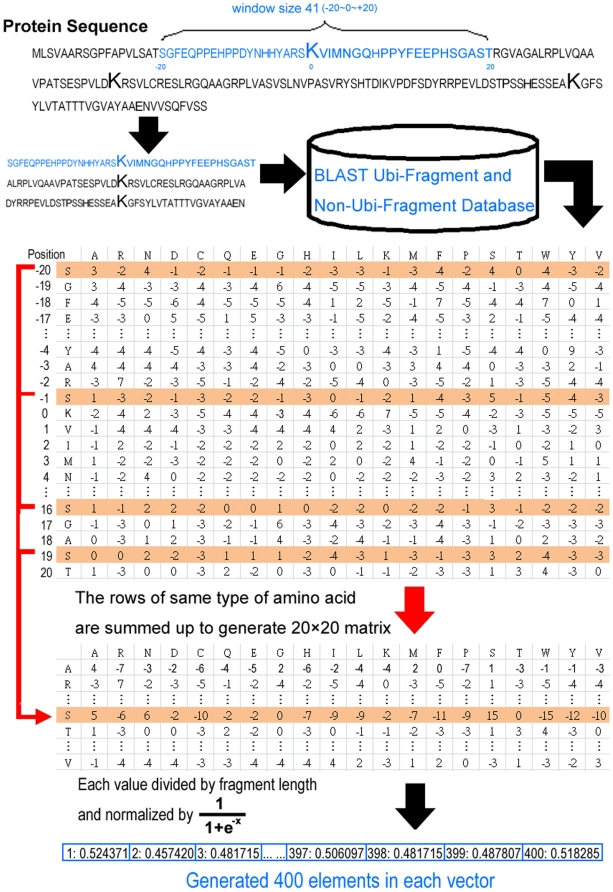
The detailed process of generating position specific scoring matrix (PSSM) and encoding the fragment of amino acid sequence by generated PSSM.

Figure 2 displays in detail how to generate the 400D PSSM features for each ubiquitylation and non-ubiquitylation site. The matrix of *m*×20 elements has rows centered on ubiquitylation or non-ubiquitylation site, extracted from the PSSM profile, where *m* represents the window size and 20 represents the position specific scores for each type of amino acid. Thereafter, the *m*×20 matrix is transformed into a 20×20 matrix by summing up the rows that are associated with the same type of amino acid. Finally, every element in 20×20 matrix is divided by the window length *m* and then is normalized using the formula: 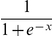.

#### Structural characteristics

Since most of the experimental ubiquitylated proteins do not have corresponding protein tertiary structures in PDB [16], an effective tool, RVP-Net [17], was used to compute the ASA value based on the protein sequence. The computed ASA value is the percentage area of each amino acid on the proteins that is accessible to the solvent. RVP-net applies a neural network to predict real ASA values of the residues based on neighborhood information, with a mean absolute error of 18.0–19.5%, defined as the absolute difference between the predicted and experimental values of relative ASA per residue [17]. Full-length protein sequences with experimental ubiquitylated sites are input to RVP-Net to compute the ASA value for all residues. The ASA values of amino acids that surround the ubiquitylation site were extracted and scaled from zero to one.

PSIPRED [18] was utilized to compute the secondary structure that surrounds the ubiquitylation sites from the protein sequence. PSIPRED is a simple and reliable method for predicting secondary structure, which applies two feed-forward neural networks to analyze the output obtained from PSI-BLAST [19]. PSIPRED 2.0 achieved a mean Q_3_ score of 80.6% across all 40 submitted target domains without obvious sequence similarity with structures that are present in PDB; accordingly, PSIPRED has been ranked as the best of 20 evaluated methods [20]. The output of PSIPRED is “H,” “E” or “C”, which stand for helix, sheet and coil, respectively. The full-length protein sequences with ubiquitylation sites are inputted to PSIPRED to determine the secondary structure of all residues. The orthogonal binary coding approach is adopted to transform the three terms that specify the secondary structure into numeric vectors. For instance, helix is encoded “100;” sheet is encoded “010,” and coil is encoded “001.”

#### F-score measurement

To study further the specificity of the substrate sites, the features that statistically differ between ubiquitylation sites and non-ubiquitylation sites are identified, based on a statistical measurement of F-score [21]. The F-score of the *i*th feature is defined as,
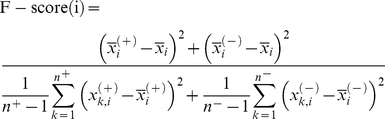
(1)where 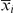, 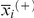 and 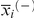 are the average value of the *i*th feature in whole, positive, and negative data sets, respectively. 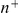 denotes the number of positive data, 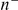 denotes the number of negative data, 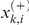 denotes the *i*th feature of the *k*th positive instance, and 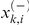 denotes the *i*th feature of the *k*th negative instance [21]. F-score supports a simple approach for measuring features that are more discriminative. If the *i*-th feature has a high F-score, then this feature effectively discriminates between positive and negative datasets.

### Training and evaluation of model

In this work, the QuickRBF package [22] has been employed to construct radial basis function network (RBFN) classifiers. As presented in Fig. S1 (See Supplementary Materials), the general architecture in an RBFN consists of three layers, namely the input layer, the hidden layer, and the output layer. The input layer broadcasts the coordinates of the input vector to each of the nodes in the hidden layer. Each node in the hidden layer then produces an activation based on the associated radial basis kernel function. Finally, each node in the output layer computes a linear combination of the activations of the hidden nodes. The general mathematical form of the output nodes in RBFN is as follows:
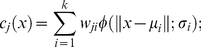
(2)where 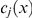 denotes the function corresponding to the *j*-*th* output node and is a linear combination of *k* radial basis functions 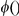 with center m*_i_* and bandwidth s*_i_*; Also, *w_ji_* denotes the weight associated with the correlation between the *j*-*th* output node and the *i*-*th* hidden node. In this work, we adopted a fixed bandwidth (σ) of five, and used all input nodes as centers (*k* = n). With its several bioinformatics applications, classification based on radial basis function network has been extensively adopted to predict factors such as the cleavage sites in proteins [23], inter-residue contacts [24], protein disorder [25], discrimination of β-barrel proteins [15], and identification of O-linked glycosylation [26].

Predictive performance of the constructed RBFN classifier is evaluated by performing *k*-fold cross validation. The original data (training data in Table 1) is divided into *k* subgroups by splitting each dataset into *k* approximately equal sized subgroups. In one round of cross-validation, a subgroup is regarded as the test set, and the remaining *k*-1 subgroups are regarded as the training set. The cross-validation process is repeated *k* rounds, with each of *k* subgroups used as the test set in turn. Then, the k results are combined to produce a single estimation. The advantage of *k*-fold cross-validation is that all original data are regarded as both training set and test set, and each data is used for test exactly once [27].The following measures of predictive performance of the trained models are defined. Precision (Pr) = TP/(TP+FP), Sensitivity (Sn) = TP/(TP+FN), Specificity (Sp) = TN/(TN+FP), and Accuracy (Acc) = (TP+TN)/(TP+FP+TN+FN), where TP, TN, FP and FN represent the numbers of true positives, true negatives, false positives and false negatives, respectively.

## Results and Discussion

### Amino acid composition of ubiquitin conjugation sites

**Figure 3:**
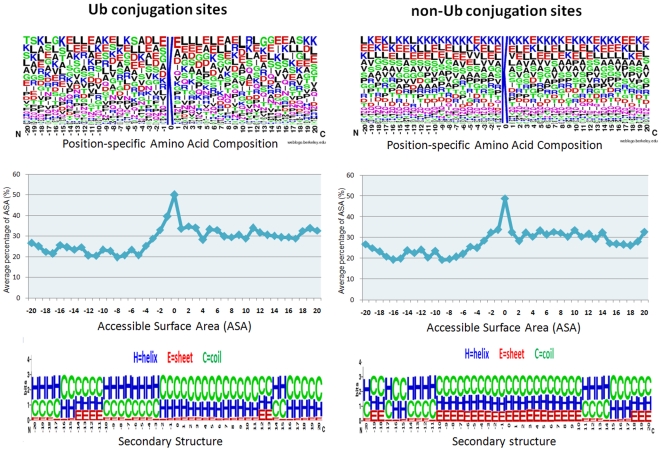
The position-specific amino acid composition, accessible surface area and secondary structure of ubiquitin conjugated lysines and non-ubiquitin conjugated lysines.

This investigation focuses on the analysis of ubiquitin conjugated lysine. In ubiquitin conjugation, the region of the ubiquitin-conjugated lysine residues is in directly contact with the E3 ligase catalytic center. Since E3 ubiquitin ligase enzymes have a substrate binding specificity, whether the region of ubiquitin-conjugated lysine conserved amino acid motifs for E3 ubiquitin ligase recognition must be explored. After the duplicated sequences of experimental ubiquitylation sites are removed, as shown in Fig. 3, the amino acids composition that flanked the ubiquitin-conjugated lysines (ubiquitylation site centered at position 0) are graphically visualized as a 41-mer sequence logo. WebLogo [28], [29] is adapted to generate the graphical sequence logo for the relative frequency of the amino acid at each position around the ubiquitylated sites. The conservation of amino acids around the ubiquitylation sites can then be easily investigated. Based on the sequence logo representation, the most abundant residues of the ubiquitylation sites are the charged and polar amino acids, including Aspartic acid (D) and Glutamic acid (E). The amino acids around the modified sites are not obviously conserved, a slight difference between the preferences of amino acids for the ubiquitylation and non-ubiquitylation sites.

Since the representation of sequence logos involves different preferences of amino acids for ubiquitylated and non-ubiquitylated sites, the statistical difference in the distribution of amino acids around ubiquitylated (Ub) and non-ubiquitylated (non-Ub) lysines is calculated. Figure S2 (See Supplementary Materials) displays the compositional differences between Ub and non-Ub sites. The more abundant amino acids at the Ub sites are Alanine (A), Aspartic acid (D), Glycine (G) and Isoleucine (I), and the depleted hydrophobic residues around these sites include Cysteine (C) and Leucine (L) around Ub sites. Moreover, the Lysine (K) and Serine (S) are less abundant around Ub sites. The amino acid sequences around the ubiquitin-conjugated sites can be alternatively grouped by various methods to generalize the sequence feature because amino acid classification is hierarchical. As presented in Table S1 (See Supplementary Materials), the three-class grouping method and the eight-class grouping method are used to 20 amino acids into subgroups that capture their chemical properties. Three-class grouping methods can be based on hydrophobicity [30], polarity [31], normalized van der Waals volume [32] and polarizability [33]. Additionally, a Two Sample Logo [34] of 41-mer compositional biases around Ub conjugation sites compared to non-Ub conjugation sites is presented in Fig. S3 (See Supplementary Materials). The amino acid residues that significantly enriched and depleted (*P*-value <0.05; t-test) around Ub conjugation sites are shown. With the investigation of position-specific difference of amino acid composition in 41-mer window length, Figure S3 indicates the positions that are distant from Ub sites have statistically significant differences of amino acid composition.

### Structural characteristics of ubiquitin conjugation sites

A side-chain of an amino acid that undergoes post-translational modification preferentially accesses the surface of a protein [35]. To investigate the preference of the solvent accessible surface area [36] that surrounds ubiquitin conjugation sites in protein tertiary structures, the experimentally identified ubiquitylation sites are mapped to the corresponding positions of the protein entries in the Protein Data Bank (PDB) [16]. The preference of the secondary structure around the ubiquitylation sites is also considered. Since most of the experimentally confirmed ubiquitylated proteins do not have corresponding protein tertiary structures in PDB [16], RVP-Net [17] and PSIPRED [18] are adopted to compute the ASA value and secondary structure, respectively, from the protein sequence. Figure 3 presents the sequence logo of the secondary structure and the average percentage of ASA in the 41-mer window (−20∼+20) of the ubiquitylation (Ub) and non-ubiquitylation (non-Ub) sites. In the investigation of secondary structure around the Ub sites, Catic *et al.* [37] has found the preference for coil structure. In this work, the observations reveal that Ub ligase (E3) prefers to recognize the regions that are located in coil (loop) or helix structures. In contrast to Ub sites, non-Ub sites don not have an obviously preferred secondary structure. In the study of solvent accessibility, most of the Ub or non-Ub lysines are located in the highly accessible surface area. However, the mean solvent-accessible surface area that surrounds the Ub sites slightly exceeds that around non-Ub sites.

### Investigation of distant sequence features for ubiquitylation sites

**Figure 4:**
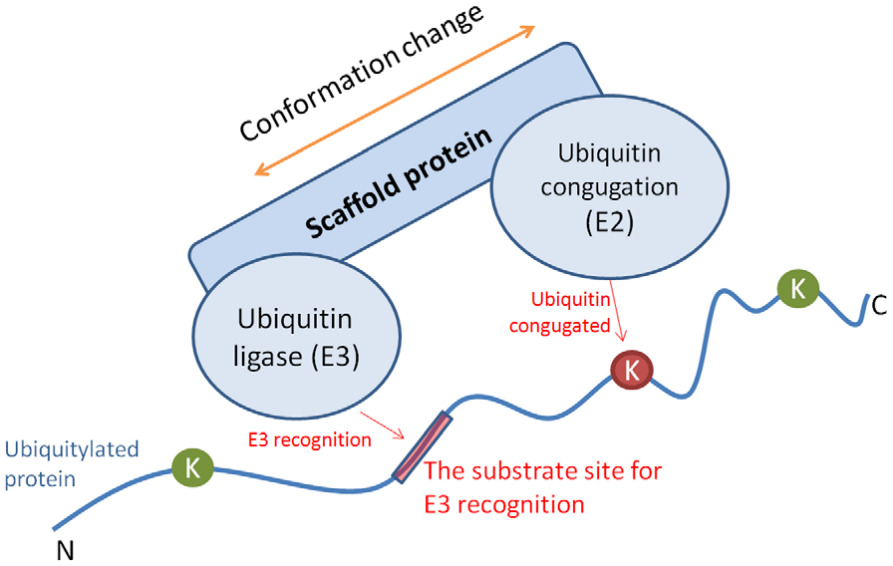
The hypothetic model of identifying the distant sequence features for E3 recognition.

**Figure 5:**
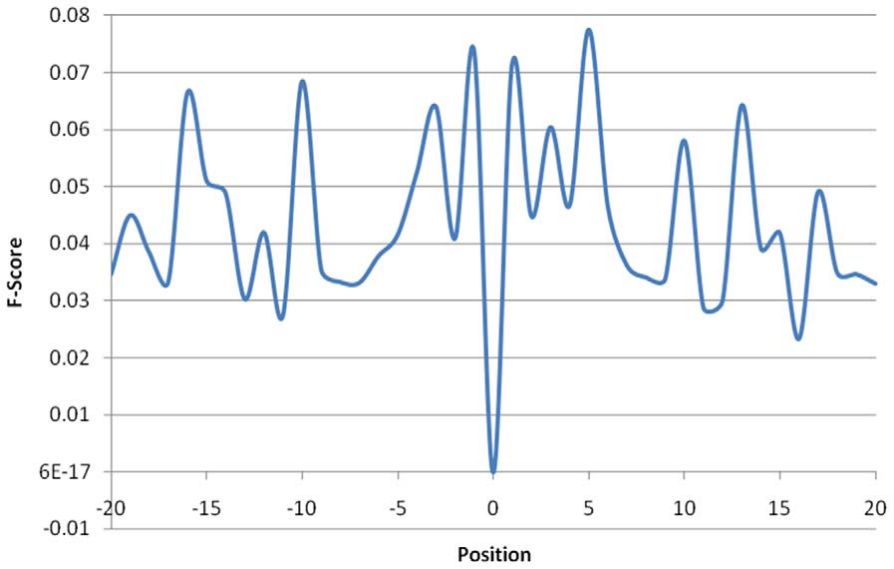
The statistically significant composition of amino acids for each position in the window length from −20 to +20. Based on the measurement of F-score, the positions −16, −10, −3, −1, +1, +5, +10, +13, and +17, containing higher value of F-score, are significant for differentiating the ubiquitylation sites from non-ubiquitylation sites.

Owing to the direct interaction between the enzyme and the substrate site, most of the proposed PTM prediction methods investigate amino acid sequences close to the modified sites. The ubiquitin conjugation pathway, which involves a sequential process with a group of enzymes known as E1 (activating enzyme), E2 (conjugating enzyme) and E3 (ubiquitin ligase) [1] motivates an investigation of the distant sequence features that are distant from ubiquitylation sites. In Fig. 4, a graphical model represents the hypothesis that contains a substrate site that is distant from ubiquitylated lysine for E3 recognition. Ubiquitin is mostly conjugated on the lysine residue of a protein by substrate recognition of Ub-ligating (E3) enzymes [2]. E3 enzymes function as the substrate recognition modules of the system and are capable of interaction with both E2 and substrate. Thus, the E3 ligase must be sufficiently specific and must act only on a defined subset of cellular targets to ensure signal fidelity [3]. Based on the measurement of the F-score in a large window size (−20∼+20), Fig. 5 displays the statistically significant composition of amino acids at positions −16, −10, −3, −1, +1, +5, +10, +13, and +17. The surrounding positions that have high F-scores are (significant for differentiating the ubiquitylation sites from the non-ubiquitylation sites. Additionally, Tables S2 and S3 (See Supplementary Materials) present the significant amino acids and di-peptides in the surrounding region (−20∼+20), which have a higher F-score value.

**Figure 6:**
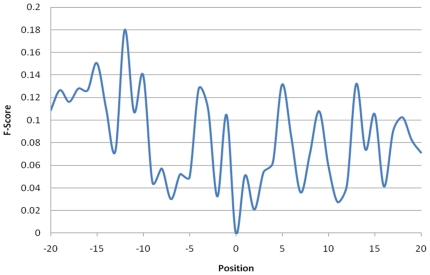
The statistically significant evolutionary information of amino acids for each position in the window length from −20 to +20. Based on the measurement of F-score, the positions −19, −17, −15, −12, −10, −4, −1, +5, +9, +13, +15 and +18, containing higher value of F-score, are significant for differentiating the ubiquitylation sites from non-ubiquitylation sites.

Position specific scoring matrix (PSSM), which is generated by PSI-BLAST [10] search against a non-redundant database of protein sequence, is utilized to obtain evolutionary information about amino acids around the ubiquitin conjugation sites. The constructed PSSM includes the probability that each amino acid is present at each position. Therefore, PSSM is regarded as a measure of residue conservation in a window of a particular length. Figure 6 displays statistically significant evolutionary information concerning amino acids at each position in the window from −20 to +20. Based on the measurement of F-scores, the positions −19, −17, −15, −12, −10, −4, −1, +5, +9, +13, +15 and +18, where the F-scores are highest, are significant for differentiating the ubiquitylation sites from the non-ubiquitylation sites. In the investigation of distant sequence features that are distant from ubiquitylation sites, the length of the training data window for learning the predictive model is set to 41-mer (−20∼+20).

**Figure 7:**
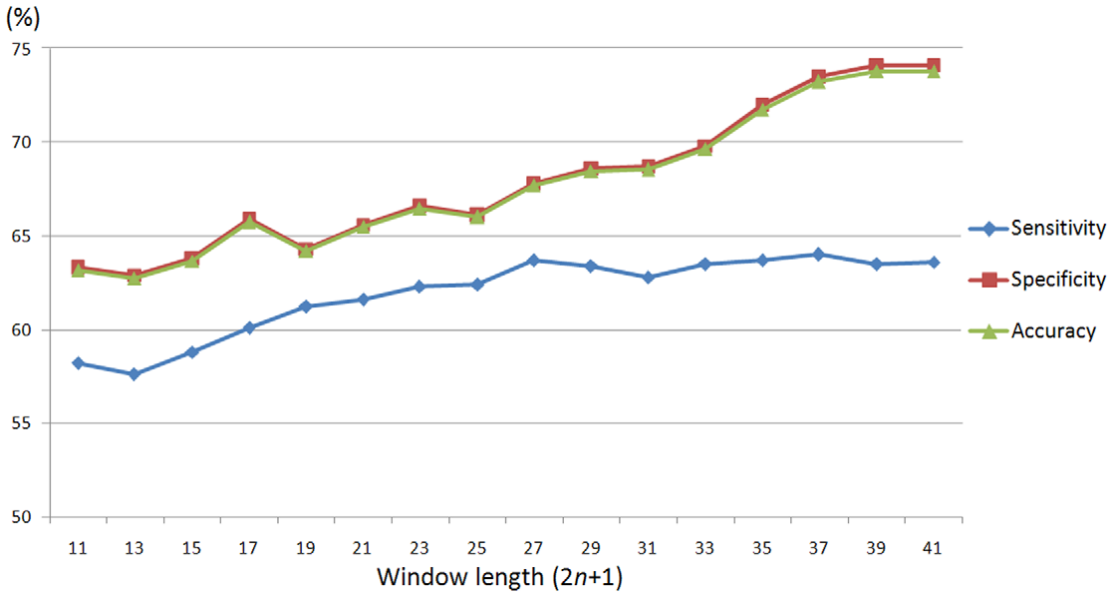
The predictive performance of the models trained with different window length varying from 11-mer to 41-mer.

To demonstrate the distant sequence features are informative for the identification of ubiquitylation sites, herein, five-fold cross-validation is performed to evaluate the models trained with various window sizes 2*n*+1, where *n* varies from five to twenty. The predictive models (RBFN classifiers) are trained with the feature of amino acid composition. Figure 7 presents the sensitivity, specificity, and accuracy of cross-validation based on various window lengths. As different window sizes from 11-mer to 41-mer are applied, the predictive accuracy improves from 63.1% to 73.7%, the sensitivity and specificity increase as well. Especially for the window length which is longer than 35-mer, the predictive power is apparently improved with the accuracy that is higher than 70.0%. As the investigation of distant sequence features in Figures 5 and 6, the model that was trained with a large window length performs better than that without the distant sequence features.

### Predictive performance of cross-validation using various training features

Most predictive models are based on the features of amino acid sequences. To determine which features can be utilized to construct models that differentiate between ubiquitylation sites and non-ubiquitylation sites, various features, including the sequence of amino acids, amino acid composition, accessible surface area, and physicochemical properties are evaluated by *k*-fold cross-validation. The amino acids (AA) and accessible surface area (ASA) around the ubiquitylated sites are encoded using a BLOSUM62 matrix and the RVP-Net-computed ASA values, respectively. Table 2 presents the predictive performance achieved using various training features, based on five-fold cross-validation. Of the models trained using individual features, those that are trained using amino acid composition slightly outperform those that are trained using amino acids, ASA, the secondary structure, or PSSMs. In particular, the model trained with the PSSM profile of non-redundant ubiquitylated protein sequences achieves an accuracy of 70%. However, the model that is trained with the secondary structure underperforms prediction based on ubiquitylation sites. According to the F-score of distant sequence features, the amino acid composition and evolutionary information (PSSM) at several flanking positions are statistically differently distributed between ubiquitylation sites and non-ubiquitylation sites. Therefore, the effects of combining informative features are evaluated. As presented in Table 2, the model that is trained using the combination of amino acid composition and PSSM profile of non-redundant fragment sequences of Ub sites performs best, with the best-balanced predictive sensitivity and specificity.

**Table 2:** The predictive performance of cross-validation using various training features.

Training features	Sensitivity (%)	Specificity (%)	Accuracy (%)
AA (Blosum62)	54.3	67.9	67.5
AA composition	63.6	74.1	73.7
AA pair composition	59.2	74.4	74.0
Accessible Surface Area	59.3	69.7	68.6
Secondary structure	58.4	59.7	59.1
PSSM 1	60.0	66.2	66.0
PSSM 2	50.9	69.3	68.7
PSSM 3	54.3	68.9	68.5
AA composition + PSSM 1	62.3	73.5	73.1
AA composition + PSSM 2	61.8	74.6	74.2
AA composition + PSSM 3	**65.5**	**74.8**	**74.5**

AA: amino acid; PSSM 1: The PSSM profiles were obtained by using PSI-BLAST against UniProt NR database; PSSM 2: The PSSM profiles were obtained by using PSI-BLAST against non-redundant Ub protein sequence database; PSSM 3: The PSSM profiles have been obtained by using PSI-BLAST against non-redundant fragments of Ub site sequ2ences.

### Predictive performance of independent testing

To determine whether the models (are over-fitted to their training data, independent sets of data concerning Ub sites and non-Ub sites are constructed and used to test the model that was trained with the combination of amino acid composition and the PSSM profile of non-redundant fragment sequences of Ub sites, which have the highest predictive accuracy. Independent test sets include 57 ubiquitylation sites and 3502 non-ubiquitylation sites, According to Table 3, are used to determine the predictive sensitivity, specificity, and accuracy of the proposed method, which were 57.9%, 72.4%, and 72.2%, respectively. Generally, the performance in an independent test approaches that of cross-validation. Whereas cross-validation outperforms independent testing, the performance of the trained model may be overestimated. The independent test establishes that the constructed RBF model does not over-fit the training data. The independent test sets were used to test other ubiquitylation predictors. The predictive sensitivity and specificity of UbiPred [8] were 52.6% and 52.6%, respectively, indicating balanced predictive performance. However, the independent test also indicates that UbiPred does not perform as well as its developers claimed. The predictive sensitivity and specificity of UbPred [9] were 42.1% and 68.7%, respectively, indicating poor sensitivity for an independent test set. In UbiPred and UbPred, the data source of experimental ubiquitylation sites is collected from UbiProt [6], which mainly stored the yeast ubiquitylation data. However, UbSite integrates the experimental ubiquitylation sites from UbiProt and UniProtKB/Swiss-Prot [7], which accumulated the ubiquitylation data from multiple species. This could partially explain the low sensitivities of UbPred and UbiPred on these independent test data which come from multiple species.

**Table 3:** Comparison between our method (UbSite) and other ubiquitylation prediction tools.

Tools		UbSite	UbiPred	UbPred
Materials		UbiProt + Swiss-Prot	UbiProt	UbiProt
Method		Radial basis function network	Support vector machine	Random forest
Training features		AAC + PSSM	Physicochemical properties	AAC + PSSM + disordered regions + physicochemical properties
Training data	Number of positive data	385	151	272
	Number of negative data	12,582	3,424	4,651
Window length		−20∼+20	−10∼+10	−12∼+12
Proposed performance	Sensitivity (%)	65.5	83.44	-
	Specificity (%)	74.8	85.43	-
	Accuracy (%)	74.5	84.44	72.0
Independent test	Sensitivity (%)	**57.9**	52.6	42.1
	Specificity (%)	**72.4**	52.6	68.7
	Accuracy (%)	**72.2**	52.6	68.3

Abbreviation: AAC, amino acid composition; PSSM, position-specific scoring matrix.

### Conclusion

This investigation proposes a method, UbSite, which incorporates the efficient radial basis function (RBF) network to identify ubiquitin conjugation sites on protein sequences. Not only the amino acid composition but also the structural characteristics, physicochemical properties, and evolutionary information of amino acids around the ubiquitylation (Ub) sites are explored. With reference to the pathway of ubiquitin conjugation, which involves a sequential process with a group of enzymes known as E1 (activating enzyme), E2 (conjugating enzyme) and E3 (ubiquitin ligase), the substrate sites for E3 recognition, which are distant from ubiquitylation sites, are examined. According to the measurement of F-score in a large window size(−20∼+20), most of the statistically significant amino acids and evolutionary information (PSSM), which can be used effectively to differentiate Ub sites from non-Ub sites, are located at large distances from Ub sites. To prevent any overestimation of predictive performance, duplicated sequences are removed using a window size determined by the collected data sets. Although the amino acid sequences around the ubiquitin conjugated sites do not contain a conserved motif, cross-validation results indicate that the integration of the evolutionary information around the sites can improve predictive performance. Table 3 compares proposed method with other ubiquitylation prediction tools, in terms of materials, method, training features, number of training data, window length, and proposed predictive performances. Furthermore, the independent test demonstrates that UbSite can outperform other ubiquitylation prediction tools.

Although the proposed method is accurate and robust, according to independent tests, some issues remain to address in the future. Firstly, the structural preferences of ubiquitin conjugation sites preferred structures at ubiquitin conjugation sites must be examined in greater detail because flanking residues are not conserved. In addition to the solvent-accessible surface area and the secondary structure, the B-factor, intrinsically disordered region, protein linker region, and other factors should be explored experimentally at ubiquitylation sites in the protein regions with PDB entries. Following work done previously on phosphorylation [38], the local 3D structure of ubiquitylation sites may be extracted for further analysis. Secondly, with reference to the pathway of ubiquitin conjugation, the ubiquitylated proteins may contain a substrate site is distant from ubiquitylation lysine and useful in E3 recognition. Therefore, the distant sequence features of ubiquitylation sites should be investigated in more detail. For instance, using the motif discovery tool, like MEME [39], to explore the significant motifs which may be the substrate sites of E3 recognition.

## Supporting Information

Figure S1 **The general architecture of RBFN consisting of input layer, hidden layer, and output layer.**
(TIF)

Figure S2 **The compositional differences of amino acids around ubiquitylation sites compared to non-ubiquitylation sites.**
(TIF)

Figure S3 **A Two Sample Logo of the compositional biases around Ub conjugation sites compared to non-Ub conjugation sites.** The amino acid residues that significantly enriched and depleted (*P*-value<0.05; t-test) around Ub conjugation sites are shown.
(TIF)

Table S1 **The graphical representation of chemical properties surrounding ubiquitylation sites using different grouping method.**
(DOC)

Table S2 **F-score of amino acid composition for 40 positions around Ubi site.**
(DOC)

Table S3 **Top 20 di-peptides with high value of F-score in the 41-mer window size (−20∼+20) around Ub site.**
(DOC)
