# Supplementary figures and images for: Incorporating Distant Sequence Features and Radial Basis Function Networks to Identify Ubiquitin Conjugation Sites
Source: PLoS One. 2011 Mar 9;6(3):e17331. doi: 10.1371/journal.pone.0017331 (PMC3052307; doi:10.1371/journal.pone.0017331)

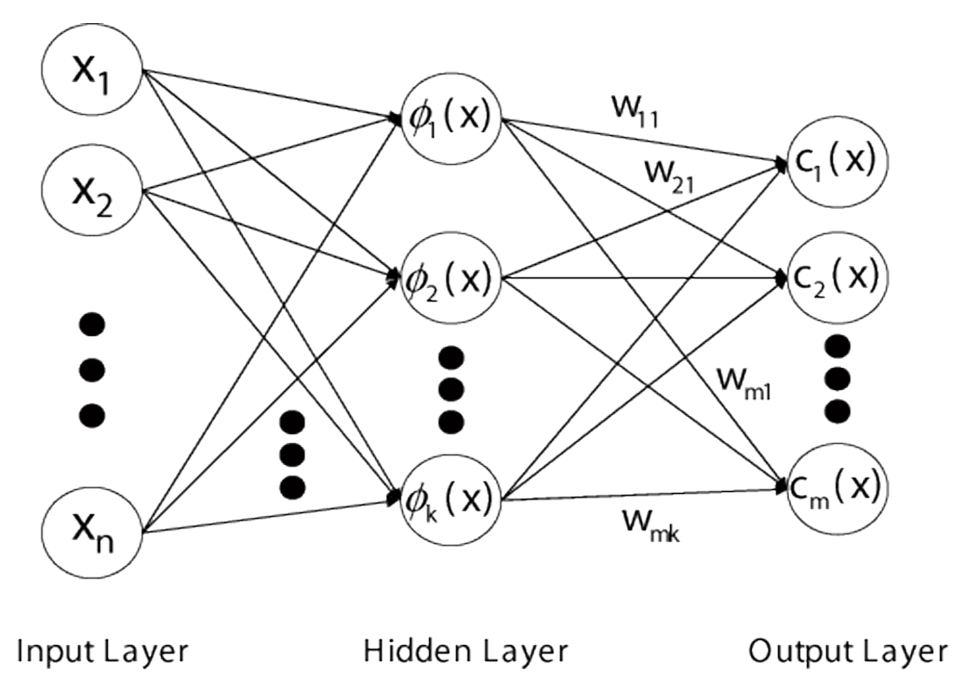

Supplement: Figure S1 — The general architecture of RBFN consisting of input layer, hidden layer, and output layer. (TIF) [file pone.0017331.s001.tif]

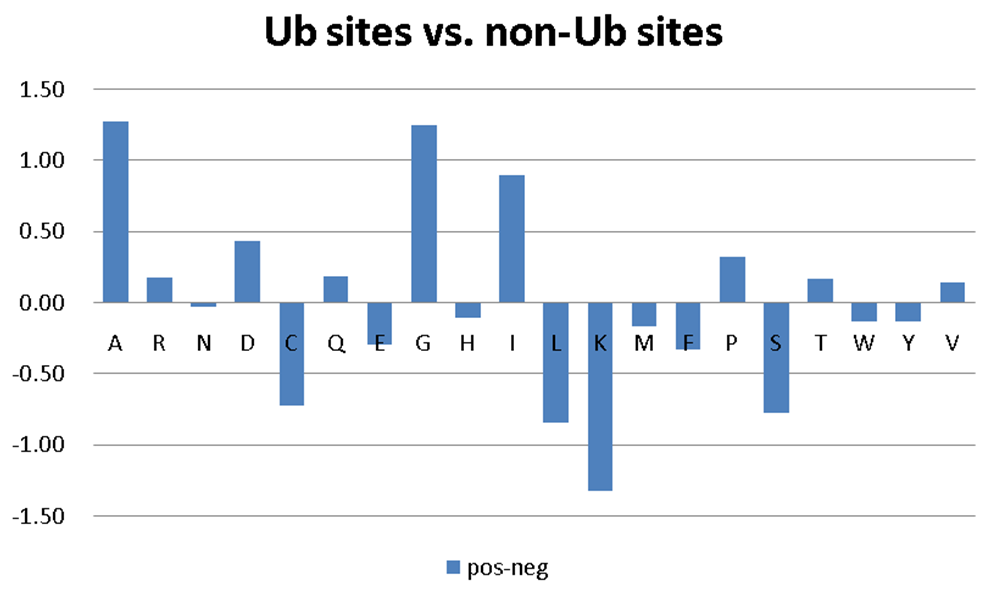

Supplement: Figure S2 — The compositional differences of amino acids around ubiquitylation sites compared to non-ubiquitylation sites. (TIF) [file pone.0017331.s002.tif]

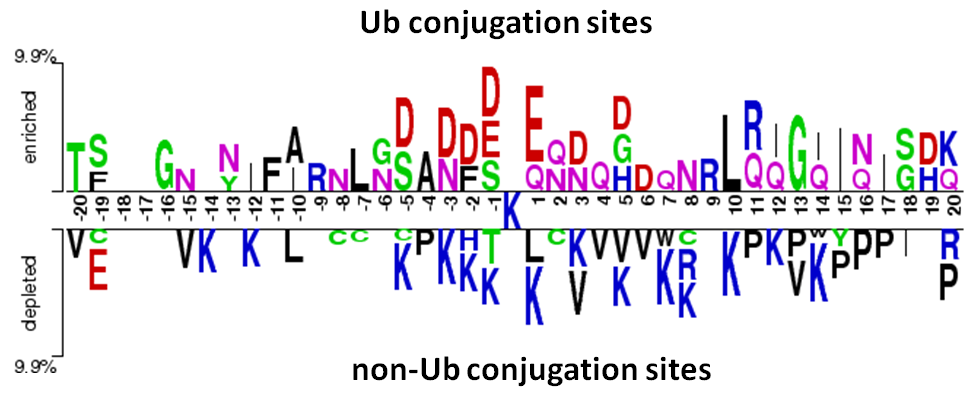

Supplement: Figure S3 — A Two Sample Logo of the compositional biases around Ub conjugation sites compared to non-Ub conjugation sites. The amino acid residues that significantly enriched and depleted (P-value<0.05; t-test) around Ub conjugation sites are shown. (TIF) [file pone.0017331.s003.tif]
