# Supplementary material for: Incorporating Distant Sequence Features and Radial Basis Function Networks to Identify Ubiquitin Conjugation Sites
Source: PLoS One. 2011 Mar 9;6(3):e17331. doi: 10.1371/journal.pone.0017331 (PMC3052307; doi:10.1371/journal.pone.0017331)
Supplement: Table S1 — The graphical representation of chemical properties surrounding ubiquitylation sites using different grouping method. (DOC) [file pone.0017331.s004.doc]

**Table S1.** The graphical representation of chemical properties surrounding ubiquitylation sites using different grouping method.

| **Grouping method** | ***No of grouped sequence** | **Subgroup** | ****Group logo** |
| --- | --- | --- | --- |
| Hydrophobicity | 194 | Polar(R) : R,K,E,D,Q,N Neutral(G) : G,A,S,T,P,H,Y Hydrophobic(C) : C,V, L, I, M, F, W | 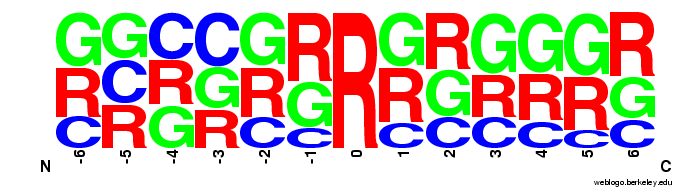 |
| Normalized van der Waals volumn | 196 | 0-2.78 (G) : G,A,S,C,T,P,D 2.95-4.0(N) : N,V,E,Q,I,L 4.43-8.03(M) : M,H,K,F,R,Y,W | 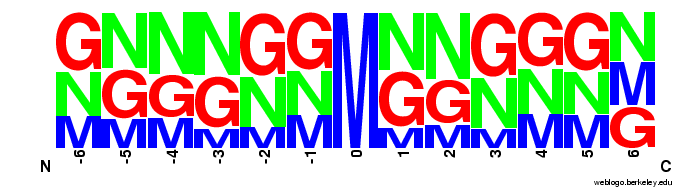 |
| Polarity | 193 | 4.9-6.2(L) : L,I,F,W,C,M,V,Y 8.0-9.2(P) : P,A,T,G,S  10.4-13.0(H) : H,Q,R,K,N,E,D | 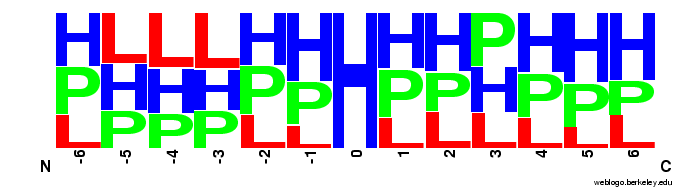 |
| Polarizability | 200 | 0-0.108(G) : G,A,S,D,T 0.128-0.186(C) : C,P,N,V,E,Q,I,L 0.219-0.409(K) : K,M,H,F,R,Y,W | 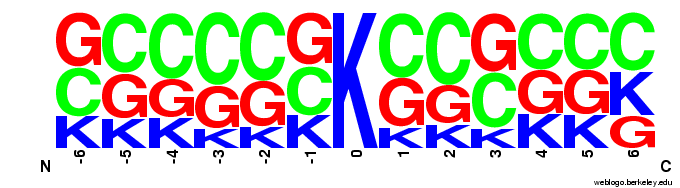 |
| 8-class | 200 | Aliphatic1(A) : A,G,P Aliphatic2(I) : I,L,V Acid(D) : D,E Base(H) : H,K,R Aromatic(F) : F,W,Y Amide(N) : N,Q Small hydroxy(S) : S,T Sulfur(C) : C,M | 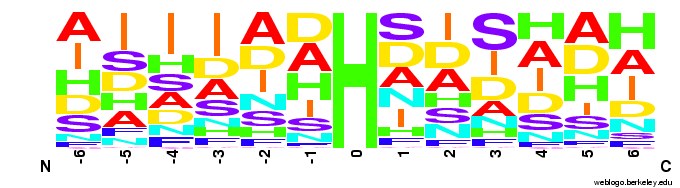 |

*each group sequence is eliminated from redundant sequence

**each symbol in the stack of group logo represent each class in the group column
