# Supplementary material for: Incorporating Distant Sequence Features and Radial Basis Function Networks to Identify Ubiquitin Conjugation Sites
Source: PLoS One. 2011 Mar 9;6(3):e17331. doi: 10.1371/journal.pone.0017331 (PMC3052307; doi:10.1371/journal.pone.0017331)
Supplement: Table S2 — F-score of amino acid composition for 40 positions around Ubi site. (DOC) [file pone.0017331.s005.doc]

**Table S2**. F-score of amino acid composition for 40 positions around Ubi site

| **Rank** | **Amino Acid** | **F-score** | **Rank** | **Amino Acid** | **F-score** |
| --- | --- | --- | --- | --- | --- |
| 1 | C | 0.053 | 11 | P | 0.003 |
| 2 | G | 0.028 | 12 | M | 0.002 |
| 3 | A | 0.027 | 13 | E | 0.001 |
| 4 | K | 0.023 | 14 | Y | 0.001 |
| 5 | I | 0.023 | 15 | Q | 0.001 |
| 6 | L | 0.014 | 16 | T | 0.001 |
| 7 | S | 0.010 | 17 | R | 0.001 |
| 8 | F | 0.006 | 18 | H | 0.001 |
| 9 | D | 0.005 | 19 | V | 0.001 |
| 10 | W | 0.004 | 20 | N | 0.000 |
