# Supplementary material for: Incorporating Distant Sequence Features and Radial Basis Function Networks to Identify Ubiquitin Conjugation Sites
Source: PLoS One. 2011 Mar 9;6(3):e17331. doi: 10.1371/journal.pone.0017331 (PMC3052307; doi:10.1371/journal.pone.0017331)
Supplement: Table S3 — Top 20 di-peptides with high value of F-score in the 41-mer window size (−20∼+20) around Ub site. (DOC) [file pone.0017331.s006.doc]

**Table S3**. Top 20 di-peptides with high value of F-score in the 41-mer window size (-20 ~ +20) around Ub site.

| **Rank** | **Amino Acid Pair** | **F-score** | **Rank** | **Amino Acid Pair** | **F-score** |
| --- | --- | --- | --- | --- | --- |
| 1 | TI | 0.044 | 11 | IF | 0.019 |
| 2 | IT | 0.035 | 12 | GV | 0.019 |
| 3 | IQ | 0.034 | 13 | QE | 0.018 |
| 4 | KL | 0.031 | 14 | FA | 0.018 |
| 5 | KK | 0.029 | 15 | AG | 0.018 |
| 6 | LK | 0.025 | 16 | CF | 0.018 |
| 7 | TL | 0.023 | 17 | EA | 0.017 |
| 8 | QF | 0.021 | 18 | IC | 0.017 |
| 9 | KI | 0.020 | 19 | SC | 0.016 |
| 10 | CE | 0.020 | 20 | AE | 0.016 |
